# Guilty bystanders: nurse-like cells as a model of microenvironmental support for leukemic lymphocytes

**DOI:** 10.1007/s10238-013-0268-z

**Published:** 2013-12-12

**Authors:** Agata A. Filip, Bogumiła Ciseł, Ewa Wąsik-Szczepanek

**Affiliations:** 1 Department of Cancer Genetics, Medical University of Lublin, Radziwillowska 11, 20-080 Lublin, Poland; 2 Department of Oncologic Surgery, Medical University of Lublin, Staszica 11, 20-081 Lublin, Poland; 3 Department of Hematooncology and Bone Marrow Transplantation, Medical University of Lublin, Staszica 11, 20-081 Lublin, Poland

**Keywords:** CLL, Nurse-like cells, Microenvironment, Apoptosis, Gene expression profiling

## Abstract

B-cell chronic lymphocytic leukemia (B-CLL) is one of the most common leukemias among the elderly and, despite many efforts, still stays incurable. Recent studies point to the microenvironment as the critical factor providing leukemic lymphocytes with pro-survival signals. Thus, the neighboring cells appear to be a perfect target for antileukemic therapy. Nurse-like cells (NLCs) largely contribute to CLL microenvironmental support. We developed the CLL lymphocyte/NLC co-culture model for the investigation of microenvironmental interactions. Viability and apoptosis were investigated in CLL lymphocytes treated with dexamethasone (DEX) and chlorambucil (CLB), with and without NLCs’ support. For the first time, the capacity of DEX and CLB to affect NLCs viability was also evaluated. Apoptosis-associated gene expression profiles of leukemic lymphocytes ex vivo and cultured with NLCs were assessed by expression arrays. CLL lymphocytes escaped spontaneous apoptosis for several months when cultured with NLCs. The presence of NLCs significantly reduced apoptosis induced with DEX and CLB (*p* < 0.001; *p* = 0.012, respectively), and their protective effect was more evident than the effect of recombinant SDF1. Both DEX and CLB also decreased NLCs viability, but to a lesser extent (mean viability in DEX-treated cultures was 37.79 % in NLCs compared to 29.24 % in lymphocytes). NLCs induced the expression of important anti-apoptotic genes in cultured CLL lymphocytes; median expression of *BCL2*, *SURVIVIN*, *BCL2A1,* and *XIAP* was significantly higher as compared to ex vivo status. The CLL lymphocyte/NLC co-culture makes up the convenient and close to the natural-state model for studying the relationship between leukemic cells and the microenvironment. Direct cell-to-cell contact with NLCs increases the expression of anti-apoptotic genes in CLL lymphocytes, thus protecting them against induced apoptosis. As the effect of antileukemic drugs is not so apparent in NLCs, the combined therapy targeted at both lymphocytes and the microenvironment should be considered for CLL patients. Simultaneous aiming at the disruption of several different signaling pathways and/or anti-apoptotic proteins may further improve treatment efficiency.

**Electronic supplementary material:**

The online version of this article (doi:10.1007/s10238-013-0268-z) contains supplementary material, which is available to authorized users.

## Introduction

B-cell chronic lymphocytic leukemia (B-CLL) is characterized by an accumulation of leukemic lymphocytes in peripheral blood, bone marrow, and lymphatic organs [1]. Increase in lymphocyte number is due both to decreased apoptosis and to slightly increased proliferation of B cells observed in proliferation centers [2]. Once isolated from circulation, leukemic cells die rapidly by apoptosis, which suggests that not only their intrinsic properties contribute to the prolonged survival. Indeed, growing evidence confirms the importance of microenvironmental signals for leukemic lymphocyte growth and resistance to the therapy [2].

CLL microenvironment is composed of cells of different origin, including activated T lymphocytes, dendritic cells, stromal cells, endothelial cells, and nurse-like cells (NLCs) [3]. The latter were named after thymic nurse cells, which were found to be necessary for proper maturation and differentiation of thymocytes [4]. CLL nurse-like cells were first described by Burger et al. in 2000 [5]. They differentiate from peripheral blood monocytes of CLL patients in in vitro cultures, but were also found in vivo, within pseudo follicles present in tissue infiltrates [6]. In vitro, NLCs protect leukemic cells against spontaneous apoptosis by producing chemokines and interleukins, i.e., SDF1, IL8, CCL2, CXCL9, and by direct cell-to-cell contact [5, 7–9]. Recently, we characterized the gene expression pattern of NLCs and stated that they resemble tumor-associated macrophages (TAMs), which support growth of solid tumor cells and thus may influence the prognosis [9].

The discovery of the role played by microenvironment in CLL development and course resulted in intensive research in this field, mainly aimed at disrupting the pro-survival signalization pathways. Many models mimicking the interactions between CLL cells and their microenvironment were proposed for this purpose. However, the cells used to date resemble CLL environment only in some aspects [10]. Here, we developed the natural model for investigation, which utilizes NLCs grown from peripheral blood of CLL patients, and we compared it with non-cell model of culture supplemented with SDF1, which is considered as the most important NLC-derived chemokine [5]. We also assessed the viability of lymphocytes cultured as such after exposure to two drugs of different mechanisms of action: dexamethasone and chlorambucil. We were the first to evaluate the sensitivity of NLCs to these antileukemic agents. Finally, we have analyzed gene expression pattern of CLL lymphocytes cultured with NLCs with special focus on anti-apoptotic genes and compared it to ex vivo status.

## Methods

### Patients

With informed consent, in accordance with the Declaration of Helsinki and approval from the Medical University Bioethics Committee, peripheral blood was obtained from 35 previously untreated patients, hospitalized at the Department of Hematooncology and Bone Marrow Transplantation, Medical University of Lublin. Five patients were excluded because less than 15 NLCs/mm^2^ was obtained in the culture. Among the remaining 30, there were 10 women and 20 men, aged 36–80 years (median 68.5), diagnosed with B-CLL according to standard criteria [11]. According to Rai classification, 5 patients were at stage 0 (16.6 %), 5 at stage I, 18 at stage II (60 %), 1 at stage III (0.3 %), and 1 at stage IV [12]. WBC ranged 10.6–530.0 × 10^9^/l (median 77.7 × 10^9^/l) (Table 1). Detailed patients’ clinical data are presented in Supplementary Table S1.

**Table 1:** Summarized clinical characteristics of CLL patients

	*n*	Mean	Median	Minimum	Maximum	SD
Age	30	63.3667	68.5000	36.0000	80.0000	14.0430
WBC (× 10^9^/l)	30	100.8900	77.7000	10.6000	530.0000	99.1000
Absolute lymphocyte count (× 10^9^/l)	30	92.3823	68.1500	7.5700	491.0000	93.7696
Absolute monocyte count (× 10^9^/l)	26	1.6126	0.6210	0.1160	14.4000	2.8841
Monocytes (%)	26	2.1719	1.0450	0.1000	10.9000	2.7014
B2 M (mg/l)	28	4.3700	3.6700	0.8000	17.6200	3.2457
LDH (IU/l)	28	364.4286	340.0000	172.0000	664.0000	123.5066
CD5/CD19 (%)	30	89.3480	91.0250	64.9100	97.1600	7.7009
CD19/ZAP70 (%)	27	21.9300	15.8400	2.6000	68.5800	18.7887
CD19/CD38 (%)	30	31.6283	23.1900	0.4400	88.4300	31.1744

*SD* standard deviation, *WBC* white blood cells count, *B2* *M* beta-2-microglobulin, *LDH* lactate dehydrogenase

### Cell isolation, culture, and analyses

PB mononuclear cells (PMBCs) were isolated by gradient density centrifugation (Lymphoprep™, AXIS-SHIELD) in glass tubes, as described elsewhere [13]. Immunophenotype was assessed ex vivo by flow cytometry with FACSCalibur apparatus equipped with CellQuest software (Becton–Dickinson Immunocytometry System) on 10,000 cells for each sample, after incubation with monoclonal mouse antihuman CD5-PE, CD19-PE-Cy5, CD38-FITC, and ZAP70 antibodies along with appropriate isotype controls (all from BD PharMingen). Antibodies were applied at 1 μg/100 μl of cell suspension (1 × 10^6^ cells in 1 % BSA/PBS), and samples were processed according to the manufacturer’s instructions. *ZAP70* gene expression was confirmed by RT-PCR using primers shown in supplementary Table S2 (DNA Gdansk). As median percentage of CD5(+)/CD19(+) lymphocytes was 91 % (Table 1), we decided not to enrich leukemic cell subpopulation for further analyses.

RNA was isolated by means of TRI reagent (Sigma), checked by spectrometry (Lambda 25 UV/VIS Spectrometer, PerkinElmer) and used for microarray analyses or for reverse transcription, followed by PCR.

CLL PBMCs (3 × 10^6^/ml) were cultured in culture flasks for adherent cells (for analyses) or on Petri dishes with grid on the bottom (squares 1 × 1 mm; Sarstedt) for counting NLCs, in RPMI 1640 with l-glutamine, supplemented with 15 % FCS and 1 × antibiotics/antimycotics solution (all from Gibco) at 37 °C, 5 % CO2. Culture medium was partially changed (1/3 vol/vol) three times a week. For statistical analyses, CLL lymphocyte suspension was decanted at day 14, and NLCs were stained with May-Grünwald Giemsa (MGG) and counted (from 50 squares of 1 mm^2^) with an Olympus CX31 microscope equipped with PLAN C 40 ×/0.65 objective.

For apoptosis induction, three versions of culture were prepared: 1. CLL lymphocytes cultured alone in the standard medium (L), 2. CLL lymphocytes co-cultured with NLCs in the standard medium (L/NLC), and 3. CLL lymphocytes cultured in the standard medium supplemented with SDF1 (L/SDF1) (rhSDF1α, 100 ng/ml, UPSTATE) [5].

Apoptosis was induced by treatment with either dexamethasone (DEX, 100nM, 30 patients) or chlorambucil (CLB, 35 μM, 8 patients) (both Sigma-Aldrich) for 24 h. The concentrations of DEX and CLB were adjusted empirically.

For analyses, NLCs were carefully washed out from lymphocytes with fresh medium, harvested by incubation with trypsin/EDTA solution (Gibco) at 37 °C for 5 min and gently scrapped with a cell-scraper (Sarstedt).

Lymphocyte and NLCs’ viability was assessed using trypan blue exclusion (TBE) assay (Trypan Blue, Sigma) and by means of flow cytometry, using fluorescein diacetate assay (FDA) (Fluorescein Diacetate, Sigma); apoptosis was examined with the Active Caspase-3 Mab Apoptosis Kit (BD PharMingen), according to the manufacturer’s protocol.

Live cell confocal microscopy was performed utilizing the Zeiss Axiovert 200 M inverted microscope with fluorescence/phase or DIC (Nomarski) imaging, fitted with 10 × and 20 × high chromatic correction objectives and PASCAL 42SP1 imaging software.

### Microarray analyses

Gene expression profiling (GEP) was assessed in CLL lymphocytes isolated ex vivo and in CLL lymphocytes cultured with NLCs for 14 days by means of expression cDNA arrays (BD Atlas Human cDNA Expression Array—Human Apoptosis Array, Clontech), as described, according to the manufacturer’s protocol, utilizing 2–5 μg DNase digested RNA per reaction [14]. Probes were synthesized using 350 mCi/l of [α-^32^P]dATP, purified by column chromatography, and the radioactivity was checked by scintillation counting. After hybridization and washing, the membranes were incubated in a phosphorimager cassette with an MS Multisensitive Storage Phosphor Screen (PerkinElmer) for 5–17 h at RT, and the image was read with a Cyclon Phosphor Imager (PerkinElmer). The results were analyzed with AtlasImage™2.7 software (BD Biosciences, Clontech). All reactions were performed in duplicate.

In order to verify the results, RNA isolated from the same samples was reverse-transcribed and PCR was carried out with primers on *BCL2*, *SURVIVIN,* and *GAPDH* genes (Supplementary Table S2). Both reactions were performed according to the manufacturer’s instructions (ImProm-II™ Reverse Transcription System, Promega and Taq PCR Core Kit, Qiagen) on 0.3 μg RNA digested with RNase-free DNase (BD PharMingen) per reaction. PCR products were assessed after gel electrophoresis with TotalLab version 1.11 Gel Analysis software.

### Statistics

Statistical analyses were performed with a free accessible R statistical package (www.R-project.org) and Statistica 10.0 PL software.

For array data analysis, the genes were filtered based on background threshold, according to the manufacturer’s instructions. The original data were then expressed in logarithmic scale and subjected to quantile normalization [15]. For comparison of pairs of groups, Welch’s corrected *t*-test was applied. The significance of diversification in the two groups was assessed by a variance analysis test (test *F*).

For other analyses, the normality of data distribution was tested by means of the Shapiro–Wilk test. Descriptive statistical analysis was performed utilizing median, minimal, and maximal values. The significance of differences between dependent samples was tested by means of the Wilcoxon matched pairs test and between independent samples by the Mann–Whitney *U* test. The strength of interdependency of two variables was expressed with Spearman’s rank correlation coefficient (*R*).

The differences were considered statistically significant with *P* values of less than 0.05.

## Results

### NLCs’ outgrowth and number

**Fig. 1:**
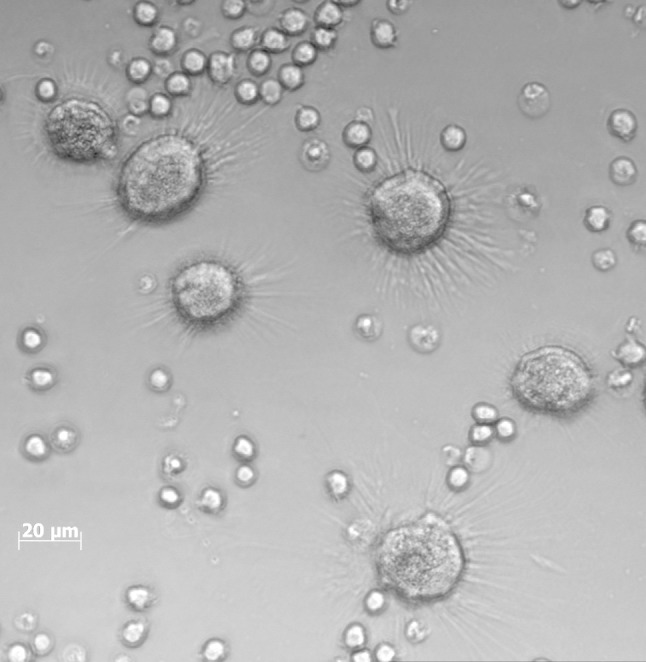
Confocal live cell imaging of CLL PBMCs culture at day 14. Cells were cultured on 3-ml Petri dishes, in standard RPMI-1640 medium supplemented with 15 % FCS and antibiotics/antimycotics. Note, large NLCs with radial projections among small, translucent lymphocytes

After 3–4 days of CLL cell culture, adherent NLCs with long projections were observed at the bottom of the culture flasks (Fig. 1). The number of NLCs increased up to the 7–8th day and then achieved a plateau. At day 14, it ranged 18–52 cells/mm^2^ (median 35) (Table 2, Supplementary Table S1). The number of NLCs positively correlated with absolute monocyte count, monocyte percentage, and β-2-microglobulin (B2 M) level (*r* = 0.45597, *r* = 0.476191, and *r* = 0.383003, respectively). No significant correlation was observed for NLC count and the stage of the disease according to Rai, WBC, serum LDH, as well as the expression of ZAP70 and CD38.

**Table 2:** Cell viability, *BCL2,* and *SURVIVIN* genes’ expression in CLL lymphocytes ex vivo and cultured with NLCs, and NLCs count at day 14

	*n*	Mean	Median	Minimum	Maximum	SD
Viability* 0 (%)	30	96.0937	96.47000	89.20000	100.0000	2.60281
BCL2** 0	30	93.9490	90.72500	46.15000	135.0000	18.68148
Survivin 0	30	20.5127	17.25500	0.00000	51.9200	15.19931
Viability 14 (%)	30	93.8843	95.60000	80.60000	100.0000	5.57868
BCL2 14	30	102.2823	98.34000	72.51000	143.8000	17.05437
Survivin 14	30	29.8413	30.94000	0.00000	69.4500	16.63554
NLC count***	30	36.2000	35.00000	18.00000	52.0000	9.51369

*SD* standard deviation

* Viability was assessed by trypan blue exclusion test

** Gene expression was assessed by RT-PCR and compared to GAPDH expression

*** NLC number per mm^2^ at day 14

### CLL lymphocytes’ viability in cultures with NLCs

**Fig. 2:**
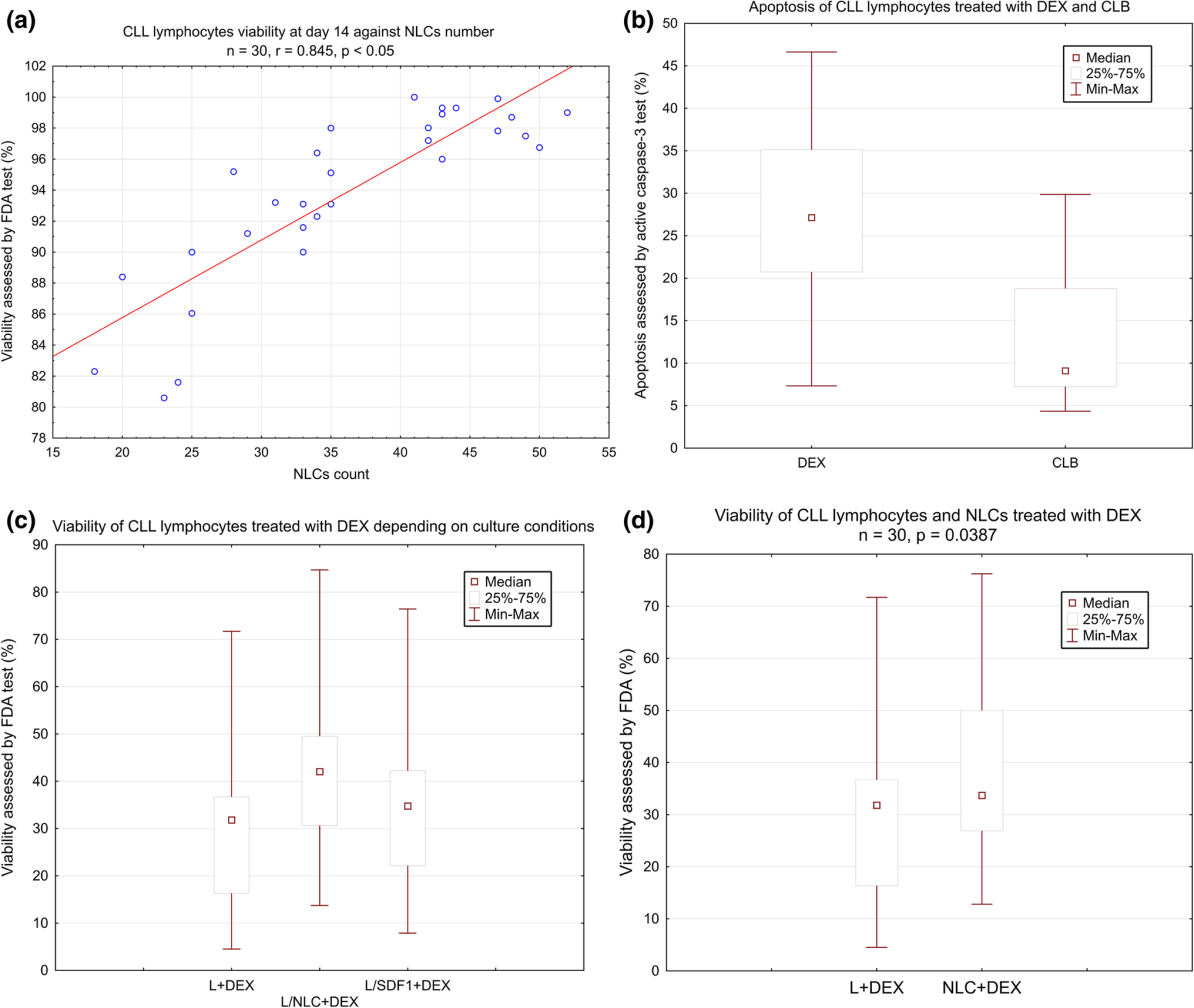
**a** Scatter diagram of CLL lymphocytes viability against NLCs number. CLL lymphocytes were cultured for 14 days as described in “Methods.” CLL lymphocyte viability was evaluated by trypan blue exclusion (TBE) assay. NLCs number was assessed on Petri dishes by counting cells from 50 squares of 1 mm^2^. **b** Apoptosis induced in CLL lymphocytes cultures by DEX and CLB, assessed by flow cytometry using active caspase-3 test. **c** The viability of CLL lymphocytes treated with DEX depending on culture conditions: L-CLL lymphocytes alone (control), L/NLC—CLL lymphocyte/NLC co-culture, L/SDF1—CLL lymphocytes supplemented with recombinant human SDF1. **d** The viability of CLL lymphocytes and NLCs treated with DEX. L + DEX—CLL lymphocytes treated with DEX for 24 h, NLC + DEX—NLCs treated with DEX for 24 h

Median lymphocyte viability assessed by TBE assay in cultures with NLCs at day 14 was 93.88 % as compared with 96.09 % at day 0, and it significantly positively correlated with the number of NLCs (*r* = 0.845075, *p* < 0.05) (Fig. 2a). While most of CLL lymphocytes died after 10 days in cultures depleted of NLCs, the CLL lymphocyte/NLC co-cultures were successfully carried out up to 14 weeks (data not shown).

### Apoptosis induced by DEX and CLB in CLL lymphocytes

**Fig. 3:**
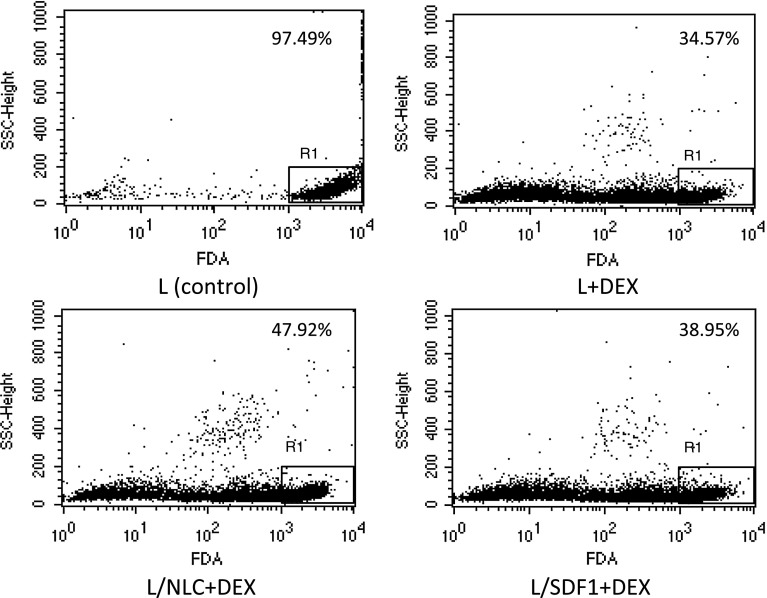
Exemplary CLL lymphocytes viability assessed by FDA in DEX-treated cultures—different variants of culture (patient # 6). FDA—fluorescein diacetate assay, L—untreated CLL lymphocytes (control), L + DEX—CLL lymphocytes treated with dexamethasone for 24 h, L/NLC—CLL lymphocyte/NLC co-culture, L/NLC + DEX—CLL lymphocyte/NLC co-culture treated with DEX for 24 h, L/SDF1—CLL lymphocytes supplemented with SDF1, L/SDF1 + DEX—CLL lymphocytes supplemented with SDF1 treated with DEX for 24 h. (see “Methods” for details). *Numbers* at the *upper right* present the proportion of viable cells

Treatment with either DEX or CLB induced apoptosis in leukemic cells as compared to control cultures (Fig. 3, Supplementary Table S3 and S4). Median lymphocyte viability assessed by FDA was lower in cultures treated with DEX or CLB than in untreated ones (31.81 vs 82.13 %, *p* < 0.0001 and 45.84 vs 79.34 %, *p* < 0.0001, respectively).

Median apoptosis assessed by caspase-3 activity test was higher in DEX-treated cultures (27.12 vs 6.15 % positive cells, *p* < 0.0001) and CLB-treated cultures (9.1 vs 6.3 %, *p* < 0.0001) as compared to control cultures (Supplementary Tables S3 and S4). DEX at a concentration of 100 nM was more efficient in inducing cell death as compared to 35 μM CLB (median viability 31.81 vs 45.84 %, respectively, *p* = 0.003923) (Fig. 2b).

CLL lymphocytes cultured with NLCs were found to be less sensitive to DEX and CLB, as compared with lymphocytes alone and lymphocytes cultured with SDF1 (Figs. 2c, 3). Median lymphocyte viability in DEX-treated cultures was 31.81 % for lymphocytes alone (L + DEX), 42.02 % for co-cultures with NLCs (L/NLC + DEX), and 34.75 % for cultures with the addition of SDF1 (L/SDF1 + DEX). The differences were significant (L + DEX vs. L/NLC + DEX, *p* = 0.000002 and L + DEX vs. L/SDF1 + DEX, *p* = 0.000148) (Fig. 2c). Similarly, in CLB-treated cultures, median viability was 45.84 % for lymphocytes alone, 53.69 % for co-cultures with NLCs and 49.66 % for cultures with SDF1 (Supplementary Table S4). In our experiments, SDF1 alone did not improve lymphocyte viability as effectively as NLCs; median viability without drugs was 82.13 % for lymphocytes alone, 82.94 % for SDF1 cultures and 88.53 % for co-cultures with NLCs (Supplementary Table S3). The difference in lymphocyte viability between the latter two culture variants was statistically significant (*p* = 0.000047).

### Apoptosis induced by DEX and CLB in NLCs

Apoptosis-inducing drugs induced decrease in viability of NLCs as well, but to a lesser extent than that of lymphocytes. Mean NLCs’ viability after DEX treatment was 37.79 %, as compared to 29.24 % of CLL lymphocytes’ viability, and the difference was statistically significant (*p* = 0.0387) (Fig. 2d, Supplementary Table S5). CLB at concentration used was less toxic than DEX; mean viability of CLB-treated NLCs was 50.66 % as assessed by FDA test.

### Gene expression profiling

After data filtering and normalization for both groups examined, the informative data concerning the expression level of 179 genes (out of 218) were obtained (Supplementary Table S6). Significant differences between CLL lymphocytes ex vivo and CLL lymphocytes cultured with NLCs concerned 36 genes (20 %), 17 genes being up-regulated at day 14. Higher expression in cultured CLL lymphocytes was observed for genes encoding anti-apoptotic proteins: BCL2, BCL2A1, SURVIVIN, XIAP, and regulators of the cell cycle, differentiation and transcription: CDK4, E2F3, CDK9, CDK5R1, MAPK3, MAPK7, and CDKN1C (Table 3). Increased *BCL2* and *SURVIVIN* expressions were confirmed by means of RT-PCR (Table 2). Moreover, using RT-PCR, we have identified three alternatively spliced *SURVIVIN* transcript variants in CLL lymphocytes: *SURVIVIN*-wt, *SURVIVIN*-2B, and *SURVIVIN*-ΔEx3 (Supplementary Figure 1).

**Table 3:** Genes up-regulated in CLL lymphocytes cultured with NLCs for 14 days compared to the ex vivo status (in descending order of mean expression values at day 0)

Gene/description	GeneBank accession #	Exp. 0* (%)	Exp. 14* (%)	Fold change	*p*
Apoptosis regulator BCL-2	M14475	88	100	1.13	0.0139
Cyclin-dependent kinase 4 (CDK4)	M14505	68	80	1.17	0.0178
CASP8 and FADD-like apoptosis regulator, CFLAR	AF010127	54	75	1.38	0.0448
E2F-3, E2F transcription factor 3	Y10479	53	74	1.39	0.0412
BCL-2-related protein A1 (BCL2A1)	U29680	47	60	1.27	0.0320
Cell division protein kinase 9 (CDK9)	L25676	42	60	1.42	0.0184
RBP2 retinoblastoma binding protein	S66431	36	63	1.75	0.0223
G2/mitotic-specific cyclin G1 (CCNG1; CYCG1)	U47413	33	58	1.75	0.0467
p53-associated mdm2 protein; MDM2	Z12020	28	45	1.61	0.0487
Extracellular signal-regulated kinase 5 (ERK5); MAPK7	U25278	27	50	1.85	0.0382
Receptor interacting serine/threonine protein kinase 2 (RIPK2)	U25994	26	40	1.53	0.0275
Apoptosis inhibitor Survivin	U75285	24	58	2.41	0.0046
TNF receptor-associated factor 5, TRAF5	U69108	22	42	1.9	0.0421
Inhibitor of apoptosis protein 3 (API3; IAP3); XIAP	U45880	18	52	2.88	0.0116
Cyclin-dependent kinase 5 activator precursor (CDK5R1)	X80343	16	49	3.06	0.0277
Extracellular signal-regulated kinase 3 (ERK3); MAP kinase 3, MAPK3	X80692	14	35	2.5	0.0323
Cyclin-dependent kinase inhibitor 1C (CDKN1C); p57-KIP2	U22398	11	33	3	0.0418

* Expression assessed by means of expression arrays

Genes down-regulated in cultured CLL lymphocytes included pro-apoptotic BCL2 family members: *BAD*, *BNIP1,* and *BNIP3*; genes coding for repair proteins: GADD45 and ERCC6; growth factor/chemokine receptors: IGFBP1, IGFBP2, and IGFBP6; genes for antioxidant enzymes: MGST12, GSTP1, and GPX1; and gene coding for death receptor FAS (Table 4).

**Table 4:** Genes down-regulated in CLL lymphocytes cultured with NLCs for 14 days compared to the ex vivo status (in descending order of mean expression values at day 0)

Gene/description	GeneBank accession #	Exp. 0* (%)	Exp. 14* (%)	Fold change	*p*
Insulin-like growth factor binding protein 2 (IGFBP2)	M35410	83	46	1.8	0.0421
Insulin-like growth factor binding protein 6 precursor (IGFPB6)	M62402	83	34	2.44	0.0365
Microsomal glutathione *S*-transferase 12 (MGST12)	J03746	72	53	1.35	0.0474
Growth arrest and DNA damage-inducible protein (GADD45)	M60974	70	48	1.46	0.0238
Insulin-like growth factor 1 (somatomedin C), IGF1	M27544	67	13	5.15	0.0312
BCL2-associated agonist of cell death, BAD	U66879	62	47	1.31	0.0121
Retinoic acid receptor epsilon (RAR-epsilon); RARB	X07282	48	29	1.65	0.0452
Cyclin-dependent protein kinase 2 (CDK2)	M68520	47	15	3.13	0.0339
Cell division cycle 25A, CDC25A	M81933	44	21	2.09	0.0386
FAS soluble protein; APO1	Z70519	44	26	1.69	0.0211
Glutathione *S*-transferase pi (GSTP1)	X15480	41	4	10.25	0.0439
BCL2/adenovirus E1B kDa interacting protein 1, BNIP1	U15172	39	7	5.27	0.0398
Cytochrome P450 reductase, POR	S90469	38	3	12.6	0.0385
Defender against cell death 1 (DAD1)	D15057	37	7	5.28	0.0486
BCL2/adenovirus E1B kDa interacting protein 3, BNIP3	U15174	33	2	16.5	0.0284
Glutathione peroxidase (GPX1)	Y00433	31	6	5.16	0.0399
Excision repair protein ERCC6	L04791	30	17	1.76	0.0437
Insulin-like growth factor 2 (IGF2); somatomedin A	M29645	27	14	1.92	0.0486
CSE1 chromosome segregation 1-like (yeast); cellular apoptosis susceptibility protein	U33286	25	8	3.5	0.0436

* Expression assessed by means of expression arrays

The Spearman’s rank correlation test demonstrated that the expression of *BCL2, SURVIVIN,* and *GAPDH* genes assessed by microarrays and RT-PCR in the same samples was comparable (*p* < 0.02).

## Discussion

Historically, CLL has been considered as an accumulative disease of lymphocytes defective in apoptosis, and this particular mechanism, but not increased proliferation, was thought to contribute toward leukemogenesis. The discovery of high spontaneous apoptosis level of CLL lymphocytes transferred to an ex vivo conditions has completely changed our conception of their intrinsic properties. In addition, telomere length studies together with proliferation assays based on deuterated water labeling pointed to the role of proliferation in lymphocyte accumulation as well [16]. Since it was not evident in peripheral blood, the thorough investigations were carried out, which unraveled dividing cells within proliferation centers of pseudofollicles located in bone marrow, spleen, and lymph nodes of CLL patients [1]. Pseudofollicle centers are composed of a mixture of small to medium and sporadically larger lymphocytes, prolymphocytes, and paraimmunoblasts [3]. They are surrounded and infiltrated by stromal cells, follicular dendritic cells, endothelial cells, and nurse-like cells. Expanded size of such proliferation centers seems to be associated with aggressive clinical course [17]. It was the second hint on the role of environment in CLL development. Recently, growing evidence confirms the significance of microenvironmental factors for CLL cell survival and proliferation. Hidden in their microenvironmental niches, leukemic lymphocytes are also resistant to therapy, which makes this issue even more important [18].

### The CLL lymphocyte/NLC co-culture model

Previously, we characterized one of the components of CLL microenvironment, nurse-like cells (NLCs) [9]. Their number in vitro correlated with serum beta-2-microglobulin, absolute monocyte count, and monocyte percentage, but not with other clinical/hematological features of CLL patients, such as stage of the disease according to Rai, lymphocytosis or CD38 and ZAP70 expression. The aforementioned observations were confirmed in the current study group.

NLCs considerably support the growth and survival of leukemic lymphocytes in vitro—in our laboratory, the co-cultures were successfully carried out for up to 14 weeks. NLCs also seem to protect leukemic cells in vivo, as we observed the tendency for longer overall survival in our patients producing less NLCs in vitro [9].

In the present study, we established the natural model for the investigation of mutual lymphocyte–environment interaction, utilizing NLCs grown from peripheral blood of CLL patients. It is easy to assemble and very convenient for studies on new therapies. Different cell culture systems mimicking CLL microenvironment have been proposed before, including murine fibroblast cells (NIH3T3) expressing CD154, murine bone marrow stromal cells (M2-10B4 line), and others [5, 10, 19]. However, these cells only in part resemble the natural cells, which compose the microenvironment.

One of the most important chemokines responsible for the enhanced survival of CLL lymphocytes is SDF1 (CXCL12), which acts via its receptor, CXCR4 [5]. SDF1 is normally produced by stromal cells within bone marrow, and it regulates B-cell development by retaining their precursors within the supportive hematopoietic microenvironment, until they are mature enough to be released into the circulation [5]. CLL lymphocytes express high levels of CXCR4, and activation of this signal transduction pathway was found to reduce spontaneous apoptosis via AKT and ERK phosphorylation [20]. Burger et al. [5] stated that NLCs were characterized by high expression of SDF1 mRNA, and synthetic SDF1 rescued CLL lymphocytes from spontaneous apoptosis. This is why we decided to use recombinant SDF1 as the control of our experiments.

Nurse-like cells exert their support not only by secreting SDF1. They enhance CLL cells survival by releasing BAFF (B-cell activating factor, CD257) and proliferation-inducing ligand APRIL (CD256) [8].

Our studies showed that NLCs might protect lymphocytes also by the secretion of IL8, the interleukin of many important functions [9]. IL8 was found to decrease CLL lymphocyte apoptosis, both spontaneous and induced by glucocorticoids [21]. Furthermore, gene expression profiling demonstrated that NLCs strongly express genes for other molecules important for lymphocyte “nursing,” such as growth factors, chemokines, cell signalization molecules, and cell adhesion molecules (i.e., CCL2, CCL8, CCL19, CXCL5, CXCL9, CX3CL1, FLT3LG, IL10, MIC3, CD44H, CD105, ICAM1, ITGAM, ITGAX, ITGB2, and SELP) [9]. High expression of genes encoding cell adhesion molecules points to the importance of direct NLC/lymphocyte contact, which in turn enables CD31–CD38 interaction, resulting in the increase in leukemic cells proliferation and migration [19, 22, 23].

The relationship between CLL lymphocytes and NLCs is clearly mutual, as the former secrete CCL3 and CCL4, which recruit NLC precursors and T lymphocytes to CLL niches [18]. In our experiments, NLCs from the cultures depleted of lymphocytes survived several days (data not shown). As the network of mutual lymphocyte–NLC interactions is so complex, the animal models that mimic the natural CLL microenvironment are far inaccurate.

Another issue important for the studies of new drugs is that NLC/lymphocyte model allows for simultaneous assessment of the effect of particular treatment both on lymphocytes and on NLCs as microenvironmental compounds.

### Apoptosis induced in the CLL lymphocyte/NLC co-cultures

Although treatment for CLL patients has dramatically changed during the last decade with the introduction of monoclonal antibodies, glucocorticoids (GCs) still are part of the therapeutic regimen, especially in fludarabine-refractory cases [24, 25].

Dexamethasone (DEX) triggers apoptosis by either transactivation through the glucocorticoid response element (GRE), transrepression of NF-kappaB, phosphorylation of intracytoplasmic tyrosine kinase RAFTK, or induction of *BCL2L11* (*BIM*) gene [26]. Moreover, since 1940 s, when GCs were introduced as first systemic therapy for CLL patients, it is known that they interfere with leukemic lymphocytes homing and redistribution between blood and secondary lymphoid tissues [27]. This brings to mind an attractive idea of combining GCs with other cytotoxic agents, which will act at leukemic cells expelled from their supportive niches.

Chlorambucil (CLB) is an alkylating agent, which impedes DNA replication and induces cellular apoptosis via the accumulation of cytosolic TP53 and subsequent activation of pro-apoptotic *BAX* gene. For many years, chlorambucil has been a standard first-line chemotherapeutic agent for patients with CLL who required treatment [11]. As orally administered, well tolerated, and inexpensive drug, it still remains an appropriate option for elderly or unfit patients [28].

We have chosen dexamethasone and chlorambucil as drugs of different mechanisms of action to induce apoptosis in CLL lymphocytes cultured in different conditions. We also wanted to evaluate whether DEX and CLB at used concentrations have any activity on NLCs.

In our study, NLCs attenuated apoptosis induced in CLL lymphocytes by both DEX and CLB (Fig. 2c). Their indirect protective effect is much more evident than the effect of recombinant SDF1, which was also observed for spontaneous apoptosis (Supplementary Table S4) [5]. This again points to the complexity of the network of pathways activated between lymphocytes and microenvironment, as well as the role of direct cell-to-cell contact [22, 23].

For the first time, the sensitivity of NLCs to antileukemic drugs was evaluated. Both DEX and CLB induced NLCs apoptosis, but the resulting decrease in their viability was not as obvious as that of lymphocytes (Fig. 2d). Given that the above observations concern the in vitro conditions, one may assume that in vivo NLCs may be even more resistant. It suggests that to achieve better response, the combined therapy, aimed both at lymphocytes and at their microenvironment, should definitely be considered.

### Changes in gene expression profiles

CLL lymphocyte gene expression in NLC co-cultures was studied by Burger and colleagues [7]. However, in our studies, we have focused strictly on the expression of genes contributing to apoptosis (Human Apoptosis Array, Clontech, 218 genes) as the process that is known to be altered in CLL. We have also selected the cases where the number of NLCs was > 15/mm^2^, to be sure that the potential gene expression changes were NLC-associated.

CLL lymphocytes cultured with NLCs demonstrated the up-regulation of genes encoding anti-apoptotic proteins, i.e., BCL2, BCL2A1, SURVIVIN (BIRC5), and XIAP (Table 3). The most important apoptosis-related gene in CLL is *BCL2* (B-cell leukemia/lymphoma). We first described in follicular lymphoma as translocated to Ig heavy chain gene locus, for many years, and *BCL2* has been considered as one of certain contributors to CLL development [29]. Its overexpression is linked to enhanced resistance to apoptosis observed in CLL lymphocytes. Deletion of 13q14, a common cytogenetic aberration in CLL, may partially account for *BCL2* up-regulation, because it involves *miR*-*15a* and *miR*-*16* loci, both being negative *BCL2* regulators [30]. *BCL2* expression may be also regulated by environmental signals. Deaglio and coworkers discovered that the expression of *BCL2* was increased in CLL lymphocytes located within proliferation centers [2]. Our results confirm that the resistance of leukemic cells to apoptosis associated with high *BCL2* expression is not solely their intrinsic feature, but is largely induced by the microenvironmental stimuli.

BCL2 itself is considered as very attractive target of the therapy in many types of cancer. First attempts with oblimersen (*BCL2* antisense phosphorothioate oligodeoxynucleotide G3139) and Oblataclax (BH3 mimetic *BCL2* inhibitor GX15-070) were not very successful in CLL [26]. The results of the phase I study of Navitoclax (ABT-263, disruptor of BCL2–BCL-xL interactions with pro-apoptotic proteins) in patients with relapsed or refractory CLL were more promising, but it caused thrombocytopenia due to BCL-xL inhibition [31, 32]. Treatment with another specific BCL2 inhibitor, ABT-199, is expected to be associated with less adverse effects [32]. However, such monotherapy still aims at only one out of many factors responsible for malignant potential of CLL cells.

*SURVIVIN/BIRC5* belongs to the inhibitor of apoptosis (IAP) family genes, which encode negative regulatory proteins that prevent apoptotic cell death. Its overexpression in cancer cells is an adverse prognostic factor, for it is associated with chemotherapy resistance, increased tumor recurrence, and shorter patient survival [33]. *SURVIVIN* expression in circulating CLL lymphocytes is low, and it was only found to be induced within proliferation centers [17]. By means of RT-PCR, we identified three alternatively spliced *SURVIVIN* transcript variants in CLL lymphocytes: *SURVIVIN*-wt, *SURVIVIN*-2B, and *SURVIVIN*-ΔEx3. The predominant one was *SURVIVIN*-wt, which was described also in ALL, AML, and B-cell lymphoma [33]. NLCs may induce *SURVIVIN* expression via activation of PI3 K/AKT pathway through BAFF and APRIL, and this up-regulation contributes to the enhanced survival of leukemic cells [8].

Another phenomenon observed in CLL lymphocytes cultured with NLCs was down-regulation of genes *BAD*, *BNIP1,* and *BNIP3*. These are BCL2 family genes encoding pro-apoptotic proteins. The higher is the ratio of anti-apoptotic BCL2 family proteins to pro-apoptotic ones, the stronger is the signal for survival. Decreased expression of *BAD*, *BNIP1,* and *BNIP3* may contribute to the resistance to apoptosis.

Cultured CLL lymphocytes demonstrated also the decrease in the expression of genes coding for antioxidant enzymes, i.e., *MGST12, GSTP1, and GPX1*. Oxidative stress is well known to play an important role in solid tumors development and response to the therapy, but there were only a few studies performed in hematological malignancies [34]. Pasanen et al. [34] described the increased expression of oxidative stress markers and antioxidative enzymes in B-cell-derived lymphomas, and its positive correlation with aggressive clinical course. As some antioxidants may also act as growth factors by inhibiting apoptosis and activating a number of transcription factors (e.g., NF-κB), the observed up-regulation may also be important for prolonged CLL cells’ survival [35].

## Conclusion

Despite many efforts, as elegantly reviewed by Hallek and by Burger, CLL still stays an incurable disease [25, 28]. The network of factors influencing proliferation and survival of leukemic cells is quite complex. Undisputedly, microenvironmental signals are crucial and should absolutely be considered in the design of new therapies. Our results proved that conventional antileukemic drugs affected microenvironment only in a small degree. Thus, the combined therapy, targeted both on lymphocytes and on the cells composing the microenvironment, should be developed. However, we have shown that NLC-derived chemokine SDF1 alone was not as effective in the protection of leukemic cells against induced apoptosis as was the presence of NLCs, which points to the involvement of other microenvironment-related factors. Hence, therapies combining conventional antileukemic drugs with disruptors of single signaling pathway, such as CXCR4 inhibitors or inhibitors of B-cell receptor-associated kinases, may not be sufficient. Strategies aimed at several different targets simultaneously should be worked out to bring an evident progress in CLL treatment. The CLL lymphocyte/NLC co-culture, as entirely comprised of the human leukemic cells, seems to be the perfect model for preliminary studies.

## Electronic supplementary material

Below is the link to the electronic supplementary material.

Supplementary material 1 (DOC 317 kb)
