# Supplementary material for: Guilty bystanders: nurse-like cells as a model of microenvironmental support for leukemic lymphocytes
Source: Clin Exp Med. 2013 Dec 12;15(1):73–83. doi: 10.1007/s10238-013-0268-z (PMC4308641; doi:10.1007/s10238-013-0268-z)
Supplement: Supplementary file 1 — Supplementary material 1 (DOC 317 kb) [file 10238_2013_268_MOESM1_ESM.doc]

Supplementary material

**Agata A. Filip^1^, Bogumiła Ciseł^2^, Ewa Wąsik-Szczepanek^3^**

**Guilty bystanders: nurse-like cells as a model of microenvironmental support for leukemic lymphocytes.**

^1^Department of Cancer Genetics, Medical University of Lublin, Radziwillowska 11, 20-080 Lublin, Poland; ^2^Department of Oncologic Surgery, Medical University of Lublin, Staszica 11, 20-081 Lublin, Poland; ^3^Department of Hematooncology and Bone Marrow Transplantation, Medical University of Lublin, Staszica 11, 20-081 Lublin, Poland.

**Table S1**

Clinical/hematological features of CLL patients and NLC count at day 14^th^

| **patient** | **sex** | **age (yrs)** | **Rai** | **WBC**  **(x10^9^/l)** | **absolute lymphocyte count**  **(x10^9^/l)** | **absolute monocyte count**  **(x10^9^/l)** | **monocytes %** | **B2M**  **mg/l** | **LDH**  **IU/l** | **CD5/CD19**  **%** | **CD5/CD19/CD38**  **%** | **CD19/ZAP70**  **%** | **NLC**  **count*** |
| --- | --- | --- | --- | --- | --- | --- | --- | --- | --- | --- | --- | --- | --- |
| 1. | F | 50 | 2 | 51 | 39,62 | 2,55 | 5 | 2,31 | 217 | 83,03 | 82,35 | ND | 47 |
| 2. | F | 58 | 2 | 75 | 66 | 5,85 | 7,8 | 3,13 | 282 | 94,33 | 65,89 | 4,62 | 49 |
| 3. | F | 79 | 1 | 53,3 | 45,3 | ND | ND | 3,37 | 277 | 90,98 | 79,97 | 5,51 | 28 |
| 4. | F | 74 | 2 | 216 | 199 | ND | ND | 5,5 | 429 | 94,36 | 5,75 | 38,49 | 43 |
| 5. | F | 36 | 2 | 90,4 | 84,98 | 1,8 | 2 | 1,52 | 234 | 90,78 | 12,54 | 15,3 | 33 |
| 6. | M | 49 | 1 | 15,1 | 11 | ND | ND | 1,23 | 172 | 75,32 | 55,82 | 28,44 | 25 |
| 7. | F | 48 | 2 | 74,2 | 70,3 | 0,28 | 0,38 | 3,97 | 345 | 89,75 | 87,21 | 23,26 | 29 |
| 8. | M | 68 | 2 | 187 | 182 | 0,616 | 0,33 | 1,98 | 218 | 92,62 | 7,86 | 27,26 | 34 |
| 9. | M | 44 | 0 | 10,6 | 7,57 | 0,154 | 1,45 | 1,68 | 366 | 79,72 | 1,06 | 12,68 | 42 |
| 10. | M | 79 | 2 | 81 | 59,78 | ND | ND | 6,38 | 664 | 90,1 | 1,38 | 4,92 | 50 |
| 11. | M | 74 | 1 | 80,4 | 73,7 | 0,601 | 0,75 | 4,58 | 414 | 90,4 | 3,58 | 2,6 | 35 |
| 12. | M | 55 | 2 | 96,7 | 92,6 | 0,17 | 0,18 | 0,8 | 508 | 95,81 | 20,18 | 6,55 | 20 |
| 13. | M | 80 | 1 | 48 | 45,8 | 0,205 | 0,43 | ND | 245 | 94,49 | 0,93 | 31,72 | 18 |
| 14. | M | 70 | 3 | 163 | 149 | 1,89 | 1,16 | 8,54 | 486 | 90,1 | 22,88 | 23,27 | 52 |
| 15. | M | 59 | 0 | 42,3 | 34,5 | 0,402 | 0,95 | 17,62 | 334 | 94,3 | 31,48 | 29,13 | 44 |
| 16. | F | 69 | 2 | 98,6 | 92 | 0,595 | 0,6 | 3,14 | 326 | 92,13 | 88,43 | 5,31 | 24 |
| 17. | M | 47 | 0 | 14,3 | 10,54 | 0,34 | 2,4 | 2,52 | 239 | 75,22 | 80,71 | 54,27 | 31 |
| 18. | M | 68 | 0 | 19,3 | 12,9 | 0,56 | 2,96 | ND | 287 | 64,91 | 26,55 | 18,96 | 43 |
| 19. | M | 69 | 2 | 46,8 | 37,89 | 3,09 | 6,6 | 3,11 | 286 | 91,07 | 0,44 | 4,53 | 41 |
| 20. | M | 60 | 1 | 27,8 | 20,49 | 1,36 | 4,9 | 7,26 | 386 | 89,57 | 64,29 | 25,39 | 23 |
| 21. | M | 71 | 4 | 179 | 177 | 0,747 | 0,42 | 2,7 | 578 | 96,84 | 37,3 | 8,65 | 35 |
| 22. | M | 37 | 2 | 90,1 | 83 | 0,641 | 0,71 | 7,47 | 514 | 89,18 | 1,18 | 12,58 | 43 |
| 23. | M | 78 | 2 | 530 | 491 | 14,4 | 10,9 | 5,44 | 555 | 96,62 | 51,05 | 64,72 | 48 |
| 24. | F | 79 | 2 | 95,1 | 86,3 | 1,74 | 1,83 | 4,09 | ND | 97,16 | 37,69 | 68,58 | 47 |
| 25. | F | 79 | 2 | 69,2 | 61,5 | 0,558 | 0,81 | 2,53 | 335 | 92,05 | 2,49 | 15,84 | 42 |
| 26. | M | 40 | 0 | 35,3 | 29,5 | 0,447 | 1,27 | 4,41 | 353 | 84,7 | 2,26 | ND | 34 |
| 27. | M | 57 | 2 | 142 | 135 | 0,87 | 0,61 | 2,78 | 299 | 79,05 | 1,48 | ND | 33 |
| 28. | M | 73 | 2 | 172 | 163 | 0,116 | 0,1 | 5,28 | ND | 97,03 | 23,5 | 47,12 | 35 |
| 29. | F | 77 | 2 | 168 | 160 | 1,32 | 0,79 | 4,84 | 480 | 92,53 | 51,03 | 5,51 | 33 |
| 30. | M | 74 | 2 | 55,2 | 50,2 | 0,626 | 1,14 | 4,18 | 375 | 96,29 | 1,57 | 6,9 | 25 |

F – female, M – male, ND – not done, WBC – white blood count, B2M – beta-2-microglobulin, LDH – lactate dehydrogenase, * - NLC number per mm^2^ at day 14^th^

**Figure S1**


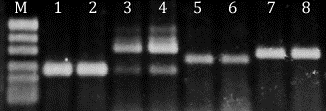


Gene expression assessed by RT-PCR in ZAP70 positive CLL patient. Note three SURVIVIN transcript isoforms. Even numbers – *ex vivo* lymphocytes, odds – lymphocytes after 14 days of culture with NLCs. 1-2. GAPDH (222bp), 3-4. SURVIVIN (SURVIVINΔex3 -224bp, SURVIVINwt – 342bp, SURVIVIN 2B – 411bp), 5-6. ZAP70 (318bp), 7-8. BCL2 (319bp)

**Table S2**

Primer sequences for RT-PCR reactions

| **primer** | **sequence (5’-3’)** | **expected length** |
| --- | --- | --- |
| *BCL2* forward | CCG CAT GCT GGG GCC GTA CAG TT | 319 bp |
| *BCL2* reverse | CGA CGA CTT CTC CCG CCG CTA CC |
| *SURVIVIN* forward | ACC GCA TCT CTA CAT TCA AG | 342bp*, 411bp**, 224 bp*** |
| *SURVIVIN* reverse | CTT TCT TCG CAG TTT CCT C |
| *GAPDH* forward | CAA CGG ATT TGG TCG TAT TG | 222 bp |
| *GAPDH* reverse | GGA TCT CGC TCC TGG AAG |

**SURVIVIN* wild type, ***SURVIVIN*2B, ****SURVIVIN*Δex3

**Table S3**

Cell viability and apoptosis in DEX treated cultures, different variants of culture

|  | **n** | **mean** | **median** | **minimum** | **maximum** | **SD** |
| --- | --- | --- | --- | --- | --- | --- |
| **cell viability assessed by FDA (%)** | | | | | | |
| **L** | 30 | 77.85300 | 82.13500 | 19.14000 | 97.87000 | 18.93120 |
| **L+DEX** | 30 | 29.24233 | 31.81500 | 4.52000 | 71.71000 | 16.79652 |
| **L/NLC** | 30 | 85.61667 | 88.53000 | 54.27000 | 98.74000 | 12.00859 |
| **L/NLC+DEX** | 30 | 42.50100 | 42.02500 | 13.74000 | 84.68000 | 16.71582 |
| **L/SDF1** | 30 | 78.82067 | 82.94500 | 26.91000 | 98.40000 | 17.82707 |
| **L/SDF1+DEX** | 30 | 34.03233 | 34.75500 | 7.88000 | 76.43000 | 16.25512 |
| **apoptosis assessed by active-caspase test (%)** | | | | | | |
| **L** | 30 | 8.67800 | 6.15000 | 0.40000 | 20.42000 | 6.82720 |
| **L+DEX** | 30 | 26.77800 | 27.12500 | 7.32000 | 46.63000 | 10.15741 |
| **L/NLC** | 30 | 5.95867 | 3.24000 | 0.28000 | 18.22000 | 5.18021 |
| **L/NLC+DEX** | 30 | 19.41633 | 21.32500 | 3.82000 | 34.78000 | 8.64710 |
| **L/SDF1** | 30 | 7.57167 | 4.58500 | 0.43000 | 19.03000 | 6.29834 |
| **L/SDF1+DEX** | 30 | 24.20200 | 25.57500 | 5.45000 | 43.21000 | 9.75796 |

SD – standard deviation, FDA - fluorescein diacetate assay, L – CLL lymphocytes alone (control), L+DEX – CLL lymphocytes treated with dexamethasone for 24 hrs., L/NLC – CLL lymphocyte/NLC co-culture, L/NLC + DEX – CLL lymphocyte/NLC co-culture treated with DEX for 24 hrs., L/SDF1 – CLL lymphocytes supplemented with SDF1, L/SDF1+DEX – CLL lymphocytes supplemented with SDF1 treated with DEX for 24 hrs.

**Table S4**

Cell viability and apoptosis in CLB treated cultures, different variants of culture

|  | **n** | **mean** | **median** | **minimum** | **maximum** | **SD** |
| --- | --- | --- | --- | --- | --- | --- |
| **cell viability assessed by FDA (%)** | | | | | | |
| **L** | 8 | 79.68500 | 79.34500 | 65.33000 | 93.20000 | 9.910218 |
| **L+CLB** | 8 | 44.71000 | 45.84000 | 33.94000 | 53.53000 | 6.791118 |
| **L/NLC** | 8 | 83.89250 | 84.45500 | 68.61000 | 95.78000 | 9.496212 |
| **L/NLC+CLB** | 8 | 52.33125 | 53.69000 | 37.84000 | 61.32000 | 8.011161 |
| **L/SDF1** | 8 | 80.21250 | 80.50500 | 65.83000 | 93.02000 | 9.095358 |
| **L/SDF1+CLB** | 8 | 47.93875 | 49.66000 | 35.92000 | 57.31000 | 7.153290 |
| **apoptosis assessed by active-caspase test (%)** | | | | | | |
| **L** | 8 | 9.18500 | 6.31000 | 2.12000 | 23.45000 | 7.861630 |
| **L+CLB** | 8 | 13.05500 | 9.10500 | 4.34000 | 29.87000 | 9.126519 |
| **L/NLC** | 8 | 6.16125 | 3.37000 | 0.68000 | 17.76000 | 6.481443 |
| **L/NLC+CLB** | 8 | 10.76250 | 7.82500 | 3.12000 | 25.15000 | 8.192193 |
| **L/SDF1** | 8 | 7.48625 | 4.79000 | 1.32000 | 19.56000 | 6.723165 |
| **L/SDF1+CLB** | 8 | 11.44750 | 7.84500 | 4.09000 | 26.89000 | 8.719652 |

SD – standard deviation, FDA - fluorescein diacetate assay, L – CLL lymphocytes alone (control), L+CLB – CLL lymphocytes treated with chlorambucil for 24 hrs., L/NLC – CLL lymphocyte/NLC co-culture, L/NLC + CLB – CLL lymphocyte/NLC co-culture treated with CLB for 24 hrs., L/SDF1 – CLL lymphocytes supplemented with SDF1, L/SDF1+CLB – CLL lymphocytes supplemented with SDF1, treated with CLB for 24 hrs.

**Table S5**

CLL lymphocytes and NLCs viability assessed by FDA test in different culture versions (%)

| **version** | **n** | **mean** | **median** | **minimum** | **maximum** | **SD** |
| --- | --- | --- | --- | --- | --- | --- |
| **L+DEX** | 30 | 29.24233 | 31.81500 | 4.52000 | 71.71000 | 16.79652 |
| **NLC+DEX** | 30 | 37.79967 | 33.69500 | 12.80000 | 76.24000 | 16.80066 |
| **L+CLB** | 8 | 44.71000 | 45.84000 | 33.94000 | 53.53000 | 6.791118 |
| **NLC+CLB** | 8 | 50.66875 | 49.78500 | 42.65000 | 62.89000 | 6.717077 |

FDA - fluorescein diacetate assay, SD – standard deviation, L+DEX – CLL lymphocytes treated with dexamethasone for 24 hrs., NLC+DEX – NLCs treated with DEX for 24 hrs., L+CLB – CLL lymphocytes treated with chlorambucil for 24 hrs., NLC+CLB – NLCs treated with CLB for 24 hrs.

**Table S6**

Mean gene expression values in CLL lymphocytes *ex vivo* and at day 14^th^ of culture with NLCs (in descending order of mean expression values at day 0)

| **gene/description** | **Exp. 0* (%)** | **Exp. 14***  **(%)** |
| --- | --- | --- |
| 23-kDa highly basic protein; 60S ribosomal protein L13A (RPL13A) | 100 | 100 |
| liver glyceraldehyde 3-phosphate dehydrogenase (GAPDH) | 99 | 99 |
| transforming protein rhoA H12 (RHO12; ARH12; ARHA) | 99 | 99 |
| c-myc binding protein MM-1 | 99 | 98 |
| growth factor receptor-bound protein 2 (GRB2) isoform; GRB3-3 | 98 | 97 |
| ubiquitin | 97 | 97 |
| induced myeloid leukemia cell differentiation protein MCL-1 | 97 | 96 |
| 40S ribosomal protein S9 | 96 | 92 |
| c-myc purine-binding transcription factor puf; NME2 | 95 | 94 |
| CDC10 protein homolog | 95 | 96 |
| cytoplasmic beta-actin (ACTB) | 94 | 95 |
| PIG7 | 94 | 93 |
| cyclin-dependent kinase 4 inhibitor D (CDKN2D); p19-INK4D | 93 | 89 |
| PDCD2 | 93 | 83 |
| glutathione-S-transferase (GST) homolog | 92 | 93 |
| chromatin assembly factor 1 p48 subunit (CAF1 p48 subunit); RBBP4 | 92 | 94 |
| caspase-8 precursor (CASP8); ICE-like apoptotic protease 5 | 91 | 86 |
| activator of apoptosis harakiri (HRK); neuronal death protein DP5 | 91 | 84 |
| HLA class I histocompatibility antigen C-4 alpha subunit (HLA-C) | 90 | 90 |
| transcription factor E2F5 | 90 | 82 |
| caspase-4 precursor (CASP4); ICH-2 protease; TX protease; ICH-2 | 89 | 91 |
| DAXX | 89 | 74 |
| apoptosis regulator BCL-2 | 88 | 100 |
| rac-alpha serine/threonine kinase (rac-PK-alpha); AKT1 | 88 | 87 |
| brain-specific tubulin alpha 1 subunit (TUBA1) | 87 | 85 |
| CD40 receptor-associated factor 1 (CRAF1) | 87 | 87 |
| growth arrest & DNA-damage-inducible protein 153 (GADD153); | 87 | 93 |
| proliferating cyclic nuclear antigen (PCNA); cyclin | 86 | 92 |
| CD27BP (Siva) | 86 | 76 |
| tumor necrosis factor receptor (TNFR) + tumor necrosis factor | 85 | 89 |
| CDC25B; CDC25HU2; M-phase inducer phosphatase 2 | 85 | 81 |
| WSL protein + TRAMP + Apo-3 + death domain receptor 3 (DDR3) | 84 | 76 |
| B-raf proto-oncogene (RAFB1) | 84 | 87 |
| insulin-like growth factor binding protein 2 (IGFBP2) | 83 | 46 |
| insulin-like growth factor binding protein 6 precursor (IGFPB-6) | 83 | 34 |
| CDC27HS protein | 82 | 86 |
| CDC37 homolog | 81 | 79 |
| Fas-activated serine/threonine (FAST) kinase | 81 | 72 |
| RBQ1 retinoblastoma binding protein | 80 | 88 |
| RBQ-3 | 80 | 78 |
| wee1Hu CDK tyrosine 15-kinase; wee-1-like protein kinase | 80 | 81 |
| p35 cyclin-like CAK1-associated protein | 79 | 82 |
| CDC16HS | 79 | 71 |
| G1/S-specific cyclin D2 (CCND2) + KIAK0002 | 78 | 88 |
| c-raf proto-oncogene | 78 | 84 |
| caspase-9 precursor (CASP9); ICE-like apoptotic protease 6 | 77 | 65 |
| p33ING1 | 77 | 73 |
| xeroderma pigmentosum group C repair complementing protein , XPC | 76 | 79 |
| BCL2 homologous antagonist/killer (BAK) | 76 | 68 |
| caspase-10 precursor (CASP10); ICE-LIKE apoptotic protease 4 | 75 | 77 |
| c-jun proto-oncogene; transcription factor AP-1 | 75 | 73 |
| NEDD5 protein homolog; DIFF6; KIAA0158 | 74 | 83 |
| dual specificity mitogen-activated protein kinase kinase 5 (MAP2K5) | 74 | 77 |
| glutathione S-transferase A1 (GTH1; GSTA1); HA subunit 1; GST2 | 73 | 85 |
| peptidyloprolyl cis-trans isomerase NIMA -interacting 1 (PIN1) | 73 | 73 |
| retinoic acid receptor beta (RXR-beta; RXRB) | 73 | 66 |
| retinoblastoma-like protein 2 (RBL2; RB2); P130 | 72 | 80 |
| microsomal glutathione S-transferase 12 (GST12; MGST1) | 72 | 53 |
| extracellular signal-regulated kinase 1 (ERK1; p44-ERK1); MAPK1 | 71 | 70 |
| caspase-2 precursor (CASP2); ICH-1 | 71 | 75 |
| cation-independent mannose-6-phosphate receptor precursor (IGF2R) | 70 | 80 |
| growth arrest & DNA-damage-inducible protein (GADD45); | 70 | 48 |
| tumor necrosis factor receptor 1 (TNFR1); | 69 | 57 |
| CDC-like kinase 2 (CLK2) | 69 | 78 |
| cell division protein kinase 4; cyclin-dependent kinase 4 (CDK4) | 68 | 80 |
| tumor necrosis factor receptor 1-associated death domain protein, TRADD | 68 | 59 |
| TNF-alpha converting enzyme (TACEA); ADAM17 | 67 | 71 |
| phospholipase A2 | 67 | 47 |
| insulin-like growth factor IA precursor (IGF1A); IGFBP1; | 67 | 13 |
| death receptor 5 (DR5); cytotoxic TRAIL receptor 2 (TRICK2A) | 66 | 68 |
| cdc2-related protein kinase PISSLRE | 66 | 69 |
| p73 (monoallelically expressed p53-related protein) | 65 | 66 |
| IGFBP complex acid labile chain | 65 | 51 |
| interleukin-1 beta convertase precursor (IL-1BC); IL-1 beta | 64 | 62 |
| ubiquitin-conjugating enzyme E2 32-kDa complementing protein | 64 | 52 |
| death-associated protein kinase 1 (DAP kinase 1; DAPK1) | 63 | 55 |
| BCL-2 associated athanogene-1 (BAG-1); | 63 | 70 |
| glutathione S-transferase theta 1 (GSTT1) | 62 | 63 |
| BAD protein; bcl-2 binding component 6 (BBC6); bcl-2L8 | 62 | 47 |
| transcription factor DP2 (Humdp2); E2F dimerization partner | 61 | 67 |
| c-jun N-terminal kinase 2 (JNK2); JNK55 | 61 | 62 |
| CDC-like kinase 3 (CLK3) | 60 | 72 |
| CD40 ligand (CD40-L); CD154 | 60 | 54 |
| apoptosis-related protein TFAR15 | 60 | 55 |
| G1/S-specific cyclin C | 59 | 64 |
| DNA fragmentation factor 45 (DFF45) | 59 | 51 |
| dual-specificity mitogen-activated protein kinase kinase 1 (MAP2K1) | 58 | 65 |
| inhibitor of apoptosis protein1 (HIAP1; API1) + IAP homolog | 58 | 41 |
| caspase-3 (CASP3); apopain precursor; cysteine protease CPP3 | 57 | 69 |
| p53-induced protein phosphatase, PPM1D | 57 | 48 |
| extracellular signal-regulated kinase 2 (ERK2); MAPK1 | 56 | 64 |
| G1/S-specific cyclin D3 (CCND3) | 56 | 39 |
| MAPK/ERK kinase kinase 3 (MEK kinase 3; MEKK3) | 55 | 59 |
| CD27 ligand (CD27LG); CD70 antigen | 55 | 60 |
| casper, a FADD- and caspase-related inducer of apoptosis (CFLAR) | 54 | 75 |
| ionizing radiation resistance-conferring protein; DAP3 | 54 | 54 |
| cell division protein kinase 5 (CDK5); tau protein kinase II | 53 | 67 |
| E2F-3 | 53 | 74 |
| cyclin H (CCNH); MO15-associated protein | 53 | 67 |
| mitogen-activated protein kinase p38 (MAP kinase p38); MAPK14 | 52 | 61 |
| NIK serine/threonine protein kinase; MAP3K14 | 51 | 53 |
| glutathione S-transferase mu1 (GSTM1; GST1); HB subunit 4 | 51 | 49 |
| apoptosis regulator BAX | 50 | 37 |
| jun-B | 50 | 56 |
| N-MYC proto-oncogene | 49 | 56 |
| TRAF6 | 49 | 41 |
| retinoic acid receptor epsilon (RAR-epsilon); RARB | 48 | 29 |
| jun-D | 48 | 30 |
| BCL-2-related protein A1 (BCL2A1); BFL1 protein | 47 | 60 |
| insulin-like growth factor binding protein 4 precursor (IGFBP4) | 47 | 46 |
| cyclin-dependent protein kinase 2 (CDK2); p33 protein kinase | 47 | 15 |
| dual-specificity mitogen-activated protein kinase kinase 6 (MAP2K6) | 46 | 36 |
| bcl-2 interacting killer (BIK); NBK apoptotic inducer protein | 46 | 43 |
| secreted apoptosis related protein 1 (SARP1) | 45 | 44 |
| hypoxanthine-guanine phosphoribosyltransferase (HPRT) | 45 | 44 |
| cell division cycle protein 25A (CDC25A) | 44 | 21 |
| FAS soluble protein; APO1 | 44 | 26 |
| apoptosis regulator BCLW; KIAA0271; BCL2L2 | 43 | 34 |
| lymphotoxin-beta (LT-beta; LTB); tumor necrosis factor C | 43 | 28 |
| cell division protein kinase 9 (CDK9); serine/threonine protease | 42 | 60 |
| c-jun N-terminal kinase 1 (JNK1); JNK46 | 41 | 40 |
| glutathione S-transferase pi (GSTP1; GST3) | 41 | 4 |
| E2F dimerization partner 1; DRTF1-polypeptide 1 (DP1) | 40 | 58 |
| proto-oncogene tyrosine-protein kinase abl; p150; c-abl | 40 | 53 |
| retinoblastoma-associated protein (RB1); PP110; P105-RB | 40 | 42 |
| serine/threonine-protein kinase PCTAIRE 1 (PCTK1) | 39 | 61 |
| BCL2/adenovirus E1B kDa interacting protein 1, BNIP1 | 39 | 7 |
| FAN protein | 38 | 25 |
| cytochrome P450 reductase | 38 | 3 |
| cyclin-dependent kinase 4 inhibitor (CDK4I; CDKN2); p16-INK4 | 37 | 37 |
| defender against cell death 1 (DAD1) | 37 | 7 |
| RBP2 retinoblastoma binding protein | 36 | 63 |
| clusterin precursor (CLU); complement-associated protein SP-40 | 36 | 45 |
| G1/S-specific cyclin D1 (CCND1); cyclin PRAD1; bcl-1 oncogen | 35 | 38 |
| retinoblastoma-binding protein 1 (RBP1) isoform I | 35 | 37 |
| cell division control protein 2 homolog (CDC2); p34 protein | 34 | 28 |
| TRAF-interacting protein (I-TRAF); TRAIP | 34 | 23 |
| G2/mitotic-specific cyclin G1 (CCNG1; CYCG1) | 33 | 58 |
| insulin-like growth factor-binding protein 3 precursor (IGFBP3) | 33 | 20 |
| BCL2/adenovirus E1B kDa interacting protein 3, BNIP3 | 33 | 2 |
| cell division protein kinase 6 (CDK6); serine/threonine protease | 32 | 50 |
| glutathione reductase (GRase; GSR; GR) | 32 | 23 |
| glutathione peroxidase (GSHPX1; GPX1) | 31 | 6 |
| protein-tyrosine phosphatase zeta precursor (R-PTP-zeta) | 31 | 11 |
| growth-arrest-specific protein 1 (GAS1) | 30 | 31 |
| excision repair protein ERCC6; Cockayne syndrome protein CSB | 30 | 17 |
| MDM2-like p53-binding protein (MDMX) | 29 | 47 |
| p53-dependent cell growth regulator CGR19; CGRRF19 | 29 | 33 |
| p53-associated mdm2 protein; MDM2 | 28 | 45 |
| nuclear factor kappa-B DNA binding subunit (NF-kappaB; NFKB) | 28 | 13 |
| extracellular signal-regulated kinase 5 (ERK5); BMK1 kinase | 27 | 50 |
| cell division protein kinase 3; CDK3 | 27 | 27 |
| insulin-like growth factor II (IGF2); somatomedin A | 27 | 14 |
| caspase-6 precursor (CASP6); cysteine protease MCH2 isoforms | 26 | 27 |
| receptor interacting serine/threonine protein kinase 2 (RIPK2) | 26 | 30 |
| G2/mitotic-specific cyclin B1 (CCNB1) | 25 | 26 |
| cellular apoptosis susceptibility protein (CAS);CSE1L | 25 | 8 |
| apoptosis inhibitor SURVIVIN | 24 | 58 |
| serine/threonine-protein kinase KKIALRE, CDKL1 | 24 | 39 |
| PIG3 | 24 | 27 |
| cyclin-dependent kinase 5 activator isoform p39I precursor (CDK5R2) | 23 | 25 |
| inducible nitric oxide synthase (INOS); type II NOS; NOS2 | 23 | 20 |
| TRAF5 | 22 | 42 |
| caspase-7 precursor (CASP7); ICE-like apoptotic protease 3 | 22 | 32 |
| inhibitor of apoptosis protein 2 (HIAP2; IAP2) + IAP homolog | 21 | 31 |
| CDC25C; M-phase inducer phosphatase 3 | 21 | 21 |
| PRB-binding protein E2F1; retinoblastoma-binding protein 3 | 20 | 38 |
| insulin-like growth factor binding protein 5 precursor (IGFBP5) | 20 | 22 |
| transcription factor NF-ATc, NFATC1 | 20 | 13 |
| G1/S-specific cyclin E (CCNE) | 19 | 33 |
| GRB-IR / GRB10 | 19 | 22 |
| serine/threonine-protein kinase PCTAIRE 3 (PCTK3) | 18 | 35 |
| inhibitor of apoptosis protein 3 (API3; IAP3); XIAP | 18 | 52 |
| extracellular signal-regulated kinase 6 (ERK6); | 17 | 29 |
| DNA-binding protein inhibitor ID-1; Id-1H | 17 | 10 |
| cyclin-dependent kinase 5 activator precursor (CDK5R1) | 16 | 49 |
| lymphotoxin-alpha precursor (LT-alpha); LTA | 16 | 5 |
| rac-beta serine/threonine kinase (rac-PK-beta); AKT2 | 15 | 6 |
| seven in absentia homolog, SIAH3, E3 ubiquitin-protein ligase | 15 | 4 |
| extracellular signal-regulated kinase 3 (ERK3); MAP kinase 3 | 14 | 35 |
| protein serine/threonine kinase STK1; cell division protein | 14 | 36 |
| fas antigen ligand (FASL); apoptosis antigen ligand (APTL) | 13 | 17 |
| secreted apoptosis related protein 3 (SARP3) | 13 | 5 |
| insulin-like growth factor I receptor (IGF1R) | 13 | 18 |
| calcium/calmodulin-dependent 3',5'-cyclic nucleotide phosphodiesterase, PDE1B | 12 | 10 |
| PIG12 | 12 | 9 |
| cyclin-dependent kinase inhibitor 1C (CDKN1C); p57-KIP2 | 11 | 33 |
| RATS1 | 11 | 24 |
| extracellular signal-regulated kinase 4 (ERK4); MAP kinase 4, MAPK4 | 10 | 16 |
| tumor necrosis factor type 2 receptor associated protein (TRAF2) | 10 | 12 |
| Abl interactor 2 (Abi-2) + Abl binding protein 3 (AblBP3) (ABI2) | 9 | 20 |
| TNF-related apoptosis inducing ligand (TRAIL); APO-2 ligand | 9 | 12 |
| cytotoxic ligand TRAIL receptor | 8 | 15 |
| I-rel (RELB) | 8 | 2 |
| phospholipase D1 (PLD 1); choline phosphatase 1 | 7 | 18 |
| p53 cellular tumor antigen, TP53 | 7 | 14 |
| caspase & rip adaptator with death domain (CRADD) | 7 | 11 |
| serine/threonine-protein kinase PLK1 (STPK13) | 6 | 19 |
| PIG10 | 6 | 3 |
| cyclin-dependent kinase inhibitor 1 (CDKN1A) | 5 | 19 |
| C-jun N-terminal kinase 3 alpha2 (JNK3A2); MAPK10 | 5 | 8 |
| PIG11 | 4 | 1 |
| CDC-like kinase 1 (CLK1) | 3 | 43 |
| cyclin G2 (CCNG2) | 3 | 16 |
| CDC6-related protein, CDC6 | 2 | 30 |
| serine/threonine-protein kinase PCTAIRE 2 (PCTK2) | 2 | 9 |
| G2/mitotic-specific cyclin A (CCNA; CCN1) | 1 | 32 |
| caspase-8 precursor (CASP8); ICE-LIKE apoptotic protease 5 | 1 | 7 |

* gene expression assessed by means of expression arrays and referred to the expression of RPL13A gene
